# Lactic Acid Bacteria Adjunct Cultures Exert a Mitigation Effect against Spoilage Microbiota in Fresh Cheese

**DOI:** 10.3390/microorganisms8081199

**Published:** 2020-08-06

**Authors:** Daniela Bassi, Simona Gazzola, Eleonora Sattin, Fabio Dal Bello, Barbara Simionati, Pier Sandro Cocconcelli

**Affiliations:** 1 Dipartimento di Scienze e Tecnologie Alimentari per una Filiera Agro-Alimentare Sostenibile (DISTAS), Università Cattolica del Sacro Cuore, 29122 Piacenza, Italy; simogazzola@libero.it; 2 BMR Genomics, srl, 35131 Padova, Italy; eleonora.sattin@bmr-genomics.it; 3 Sacco s.r.l., 22071 Cadorago, Italy; F.DalBello@saccosrl.it; 4 EuBiome Srl, 35129 Padova, Italy; barbara.simionati@gmail.com

**Keywords:** lactic acid bacteria, adjunct cultures, cheese, spoilage, Gram-negative bacteria, mitigation, shelf life

## Abstract

Lactic acid bacteria (LAB) have a strong mitigation potential as adjunct cultures to inhibit undesirable bacteria in fermented foods. In fresh cheese with low salt concentration, spoilage and pathogenic bacteria can affect the shelf life with smear on the surface and packaging blowing. In this work, we studied the spoilage microbiota of an Italian fresh cheese to find tailor-made protective cultures for its shelf life improvement. On 14-tested LAB, three of them, namely *Lacticaseibacillus rhamnosus* LRH05, *Latilactobacillus sakei* LSK04, and *Carnobacterium maltaromaticum* CNB06 were the most effective in inhibiting Gram-negative bacteria. These cultures were assessed by the cultivation-dependent and DNA metabarcoding approach using in vitro experiments and industrial trials. Soft cheese with and without adjunct cultures were prepared and stored at 8 and 14 °C until the end of the shelf life in modified atmosphere packaging. Data demonstrated that the use of adjunct cultures reduce and/or modulate the growth of spoilage microbiota at both temperatures. Particularly, during industrial experiments, *C. maltaromaticum* CNB06 and *Lcb. rhamnosus* RH05 lowered psychrotrophic bacteria of almost 3 Log CFU/g in a 5-week stored cheese. On the contrary, *Llb. sakei* LSK04 was able to colonize the cheese but it was not a good candidate for its inhibition capacity. The combined approach applied in this work allowed to evaluate the protective potential of LAB strains against Gram-negative communities.

## 1. Introduction

Food spoilage is considered an important factor affecting the postharvest food losses, defined as the amount of edible food that is available for human consumption but is not consumed for any reason. The dairy products are the third food category in terms of the estimated total value of food losses and waste at retail and consumer levels. Gustavsson et al. [1] estimated that more than 20 and 12% of the initial milk and dairy production is lost or wasted respectively in the US and in Europe, and most of these losses occur at processing or consumption stages [2]. The main causes of cheese spoilage are microorganisms (bacteria, yeasts, and molds) that can grow during the manufacturing process or all along the shelf life, modifying sensorial properties, aroma, structure, and color of foodstuffs. While clostridia are the main cause of spoilage in long ripened hard cheeses [3,4], in fresh cheeses, psychotropic Gram-negative bacteria are the main component of the spoilage microbiota [5,6,7,8], often causing gas production and paste defects with slime production and unpleasant taste. Few recent studies addressing fresh cheese defects by spoilage bacteria communities in the processing environments have been published [9,10] using a culture-independent approach based on the high-throughput sequencing of 16S and 26S rRNA amplicons. The core microbiota found in the different analyzed samples was dominated by lactic acid bacteria and by members of the genera *Pseudomonas*, *Acinetobacter*, and *Psychrobacter*. Moreover, the increasing demand of fresh cheeses with reduced salt content raises the risk of higher contamination by this kind of spoilers as assessed in fresh cheese with a reduction of NaCl concentration to 1.3% which resulted in an increased growth of the spoilage bacterium *P. fragi* [11].

The strategy to use lactic acid bacteria (LAB), added as starter or adjunct cultures, represents an effective approach to reduce the undesirable microorganisms in foods, due to their ability to produce different antagonistic substances such as bacteriocins and antimicrobial peptides, hydrogen peroxide, and organic acids [12,13,14]. In the last decade, several studies were focused on the use of LAB in dairy productions to control the growth of pathogenic bacteria such as *Listeria monocytogenes* [15,16], Shigatoxin-producing *Escherichia coli* [17], and *Salmonella* [18]. An increasing number of studies have demonstrated that bacteriocin-producing LAB are able to inhibit spoilage bacteria in vitro and in cheese experiments and that they can be considered as an appealing alternative to chemical preservatives [19]. LAB with protective potential are used alone or as a pool, like *Lacticaseibacillus rhamnosus* which was demonstrated to limit the fungal spoilage in cottage cheese alone or in combination with *Bifidobacterium animalis* subsp. *lactis* [20], or a pool of six strains of the *Lactobacillus* genus were found to significantly delay the growth of aspergilli on the cheese surface [21].

In this study, the main objectives were: (i) to characterize the spoilage microbiota of a fresh soft cheese with defect and to identify the causative agents, using a combination of cultivation-based methods and the next generation sequencing of 16S rRNA bacterial amplicons; (ii) to select adjunct cultures of LAB able to inhibit the strains responsible for the spoilage; and (iii) to assess, under industrial conditions, how adjunct cultures, selected for their antagonism capacity against the spoilage bacterial species, can modify the cheese bacterial community and reduce the occurrence of spoilage and packaging blowing.

## 2. Materials and Methods

### 2.1. Bacterial Strains and Culture Conditions

Bacteria used in this study are listed in Table 1.

Lactobacilli were grown in anaerobic condition at 37 °C for 48 h in MRS broth at pH 6.5 (Becton, Dickinson and Company, Franklin Lakes, NJ, USA), *Carnobacterium* in anaerobic condition at 37 °C for 48 h in MRS broth at pH 8.5, and the Gram-negative bacteria in aerobic condition at 30 °C for 24 h in LB broth medium (Oxoid, Hampshire, UK).

### 2.2. Sampling and Microbiological Analyses

Fresh cheeses with 0.65% salt content stored at 8 °C for 25 days, one week after the usual shelf life, were collected from a dairy farm. Microbiological analyses were made on 15 batches produced in summer and 15 during autumn, collecting three cheeses from each production. Samples were processed as follows: 10 g of cheese were homogenized with 90 mL of 2% sodium citrate in a Stomacher Lab-Blender 400 (Laboratory Blender Seward, London, UK), tenfold diluted in peptone water, and plated on different selective media. Plate counts were done for the following microorganisms using different selective media: staphylococci on mannitol salt agar (Oxoid, Hampshire, UK) at 37 °C for 24 h, enterococci on Slanetz & Bartley medium (Oxoid, Hampshire, UK) at 37 °C for 24 h, heterofermentantive lactobacilli on MRS (Merck KGaA, Darmstadt, Germany) added with 8 µg/mL of vancomycin (Sigma-Aldrich, St. Louis, MO, USA) in anaerobic condition at 37 °C for 48 h, *Pseudomonas* on *Pseudomonas* agar (Oxoid, Hampshire, UK) at 30 °C for 24 h, Enterobacteriaceae on violet red bile agar (Oxoid, Hampshire, UK) at 30 °C 24 h, and *Streptococcus thermophilus* was evaluated using M17 agar (Oxoid, Hampshire, UK) under aerobic conditions at 45 °C for 24 h. Finally, yeasts and molds were plated on Rosa Bengala (Oxoid, Hampshire, UK) and YEPD medium at 30 °C for 48 h.

### 2.3. DNA Fingerprinting and Taxonomical Identification

DNA was extracted from selected colonies from all media using Microlysis (Clent Life Science, Amblecote Stourbridge, UK). RAPD PCR typing method was performed with RAPD2 primer (5′-AGCAGCGTGG-3′) as described by Cocconcelli et al. [22] and REP PCR was carried out with GTG5 primer (5′-GTG GTG GTG GTG GTG-3′) as described by Woods et al. [23]. PCR was performed in Mastercycler EPgradient S thermocycler (Eppendorf, Hamburg, Germany). PCR products were separated by electrophoresis at 100 V in a 2% agarose gel and stained with Syber Safe (Invitrogen, Carlsbad, California, US). The 16S rRNA gene was amplified using the primers P1 (5′-GCGGCGTGCCTAATACATGC-3′) and P6 (5′-CTACGGCTACCTTGTTACGA-3′). P1 was used to determine the partial 16S rRNA gene sequence. The 16S rRNA gene sequences were compared with the sequences present in the small sub-unit database (SSU-Prok) of Ribosomal Database Project Release 10 [24]; to obtain taxonomical and similarity rank calculations (S_ab) [25].

### 2.4. Inhibition Assays

A total of 14 adjunct cultures were tested for their inhibitory activity using MRS medium and fresh cheese agar (FCA) (Table 1). FCA was composed by 120 g/L of fresh cheese and 20 g/L of agar. After homogenization, the medium was treated at 90 °C for 3 min to melt and pasteurized the medium. Adjunct cultures (10^7^ CFU/mL) were inoculated in the agar media before pouring the plates, then, decimal dilution of the spoilage strains (10 µL) were spotted over the agar surface and plates incubated in aerobic condition at 8 °C and 14 °C for 48 h. As control, MRS and FCA plates without adjunct cultures but containing *S. thermophilus* ST022 (Clerici-Sacco Group, Como, Italy), as the solely starter culture, were used. The inhibitory activity was detected by assessing the count reduction of the spoilage strains on the media containing the adjunct cultures when compared with the control.

### 2.5. Industrial Trial

The studied cheese is a fresh “primosale” cheese, made with low fat pasteurized cow milk. In brief, the cheese-making process was the following: milk was pasteurized for 30 s at 78 °C, coagulation occurred at 37 °C after the addition of rennet, the curd was molded and then brined by immersion in a saturated salt solution (0.65% final salt concentration). Cheese was packaged in a modified atmosphere (70% N_2_ and 30% CO_2_) and stored at 4 °C. The final pH of the cheese was 5.8. The industrial trial was based on three subsequent production cycles of fresh cheeses, used as replicates, and manufactured in the same cheese plant.

The experimental design was as follows: a control (STD) made using *S. thermophilus* ST022 as a starter culture was inoculated at a dose of 10^7^ CFU/mL of milk. Treated cheeses, manufactured as the control, were produced by adding 5 × 10^6^ CFU/mL of each different adjunct cultures, *Llb. sakei* LSK04 (SA), *Lcb. rhamnosus* RH05 (RH), *C. maltaromaticum* CNB06 (CB) (Clerici-Sacco Group, Como, Italy) in single or a mix of them (MX) where the three strains were present in equal amounts. Each treatment was manufactured in parallel in a different vat. In each replicate, half of the production was used for a challenge study, where cheeses were spiked, just before the packaging, by spraying onto the cheese surface a mixture of 10 spoilage isolates (Table 1) with a final concentration of 1 × 10^2^ CFU/cm^2^. Spiked samples were named respectively STD1, SA1, RH1, CB1, MX1. Cheese samples packaged under a modified atmosphere composed by 70% N_2_ and 30% CO_2_ (MAP), were stored for 5 weeks at 8 and 14 °C, simulating, in the second case, a thermal abuse.

### 2.6. DNA Extraction

DNA was extracted from cheese samples according to the following protocol: 200 mg of cheese paste were collected in triplicate, processed with sodium citrate 2%, incubated for 10 min at 45 °C, and centrifuged at 8000 rpm for 8 min. Then, 40 µL of proteinase K and 400 µL of lysis buffer were added to the cell pellet and the tubes were then incubated at 56 °C with gentle shaking for 2 h. DNA extraction was performed using the Spin Tissue Mini Kit Invisorb (Invitek, Berlin, Germany) following the manufacturer’s instructions. The elution step was repeated twice to increase the final DNA yield.

### 2.7. 16S rRNA Amplicon Library Construction, Sequencing, and Bioinformatics

The V3-V4 regions of 16S rRNA gene were amplified using 331F primer: 5′-TCCTACGGGAGGCAGCAGT-3′ and 797R: 5′-GGACTACCAGGGTATCTAATCCTGTT [26]. Primers were modified with forward overhang: 5′-TCGTCGGCAGCGTCAGATGTGTATAAGAGACAG -[locus-specific sequence]-3′ and with reverse overhang: 5′- GTCTCGTGGGCTCGGAGATGTGTATAAGAGACAG-[locus-specific sequence]-3′, necessary for dual index library preparation. After indexing and pooling steps, libraries were loaded on Illumina MiSeq (Illumina Inc., San Diego, CA, USA) and sequenced with 2 × 300 bp paired end approach. Sequencing reads filtered for average quality (Q > 30) and R1 and R2 were merged using FLASH with default parameters [27]. Biological replicates were pooled. Qiime v 1.9.1. [28] was used to perform the full analysis from OTU picking to the statistical analysis, using the pick_closed_reference_otus wrapper for OTU picking with UCLUST against RDPII database trainset14_032015. OTUs were filtered at 0.005% abundance [29] and representative sequences were identified using SeqMatch ver.3 release 11, retaining the best KNN match. Alpha and beta diversity were studied by means of QIIME 1.9.1 using the alpha_diversity and beta_diversity_through_plots wrappers. Composition and diversity were analyzed using MicrobiomeAnalyst softwares [30]. PICRUSt (Phylogenetic Investigation of Communities by Reconstruction of Unobserved States, http://picrust.github.io/picrust) was used to predict the functional profiles of the microbial communities. QIIME 1.8 was used to pick the OTUs at 97% identity against the Greengenes database (version 05/2013) with the closed reference method. Statistical Analysis of Metagenomic Profile software package (STAMP) was used for the statistical analysis. Significant differences among all samples were calculated using ANOVA test along with Tukey–Kramer test with p values (*p* < 0.05) [31].

#### Nucleotide Sequence Accession Numbers

Reads were deposited in the Sequence Read Archive (SRA) database under accession number SRP071740.

## 3. Results

### 3.1. Characterization of Fresh Cheese Spoilage Microbiota by Culture-Dependent Approach

**Figure 1:**
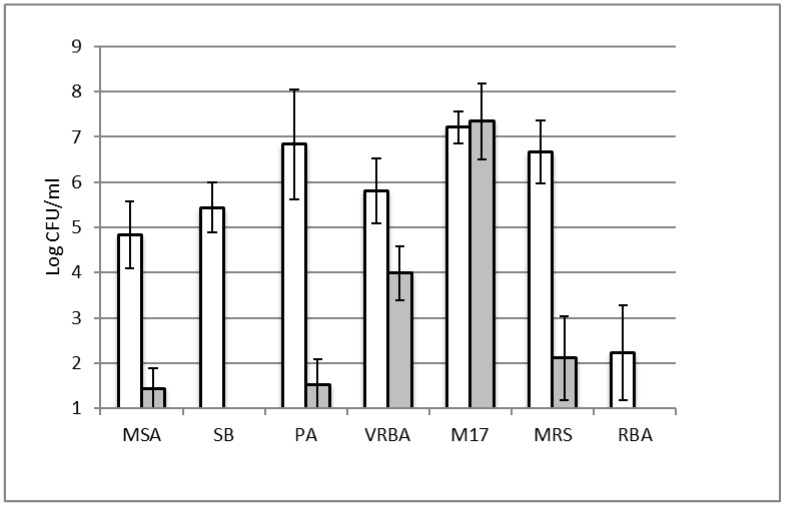
Microbiological counts for the considered bacterial groups in summer (white bars) and autumn (grey bars) cheese productions. MSA mannitol salt agar for staphylococci, SB Slanetz & Bartley medium for enterococci, PA Pseudomonas agar for *Pseudomonas*, VRBA violet red bile agar for *Enterobacteriaceae*, M17 agar for *S. thermophilus*, MRS De Man Rogosa and Sharpe agar for heterofermentantive lactobacilli, RBA Rosa Bengala for yeasts and molds.

The microbiological counts performed on samples collected one week after the end of the shelf life showed significantly higher counts in summer batches than in cheese manufactured during the autumn, for all the considered bacterial groups, with the only exception of *S. thermophilus*, which was present in comparable numbers, being used as starter culture in both periods, as shown in Figure 1.

This observation was consistent with the notice that summer samples, after a prolonged shelf life (one week after expiration date), presented a higher incidence of surface smear and gas defects (data not shown). In particular, the spoilage microbiota of summer production was dominated by bacteria growing on selective media for *Pseudomonas* (6.9 Log CFU/g), heterofermentative *Lactobacillus* (6.7 Log CFU/g), and *Enterobacteriaceae* (5.8 Log CFU/g), while other bacterial groups were found in lower numbers. To better identify the spoilage microbiota, 200 randomly selected colonies from agar plates of the different media and from both seasons were analyzed by RAPD-PCR and REP-PCR to determine the prevalence of single spoilage strains in the cheese products and the possible persistence in the production environment. RAPD and REP-PCR fingerprinting analysis recognized 48 different strains that were subsequently identified by 16S rRNA gene sequence analysis (Table 2).

The complex microbial community of sampled cheeses was dominated by *Obesumbacterium proteus*, which was detected on both VRBA and Pseudomonas agar plates. The most prevalent strains of this species were isolated from both summer and autumn manufactures (Table 2), suggesting their persistence in the cheese plant. In lower numbers, also strains belonging to *Aerococcus urinaeequi* and *Enterococcus faecalis* have been found in the cheese samples from summer production. In addition, different species of *Pseudomonas* genus were detected: *Pseudomonas libanensis*, *P. fragi*, and *P. psychrophila* uniquely identified in the summer samples while *P. brennerii*, *P. gessardii*, and *P. proteolytica* differently found in autumn’s cheeses.

### 3.2. In Vitro Selection of Adjunct Cultures

The second purpose of this study was to ascertain if adjunct cultures of LAB were able to reduce the detected spoilage defect in fresh cheese. To identify the most suitable cultures, 14 different strains (Table 1), known for their ability to produce antimicrobial compounds, were evaluated (data not shown). These strains were tested on agar plates and in cheese agar models against ten strains isolated from the spoiled fresh cheeses object of this study (Table 1). Results showed that the most active strains at the two temperatures of incubation (8 and 14 °C) were *Llb. sakei* LSK04, *Lcb. rhamnosus* LRH05, and *C. maltaromaticum* CNB06 on both used media (MRS and FCA). These cultures induced a reduction of more than 3 Log CFU when compared to the control strain. Additionally, the three selected strains were tested against each other to assess the possibility of cross inhibition when used together in mixed culture. No evident effect of cross inhibition was detected, being the reduction of viable cells below 1 Log CFU. Consequently, these three strains were selected for further studies in industrial conditions.

### 3.3. Efficacy Assessment of Adjunct Cultures in Industrial Conditions

**Figure 2:**
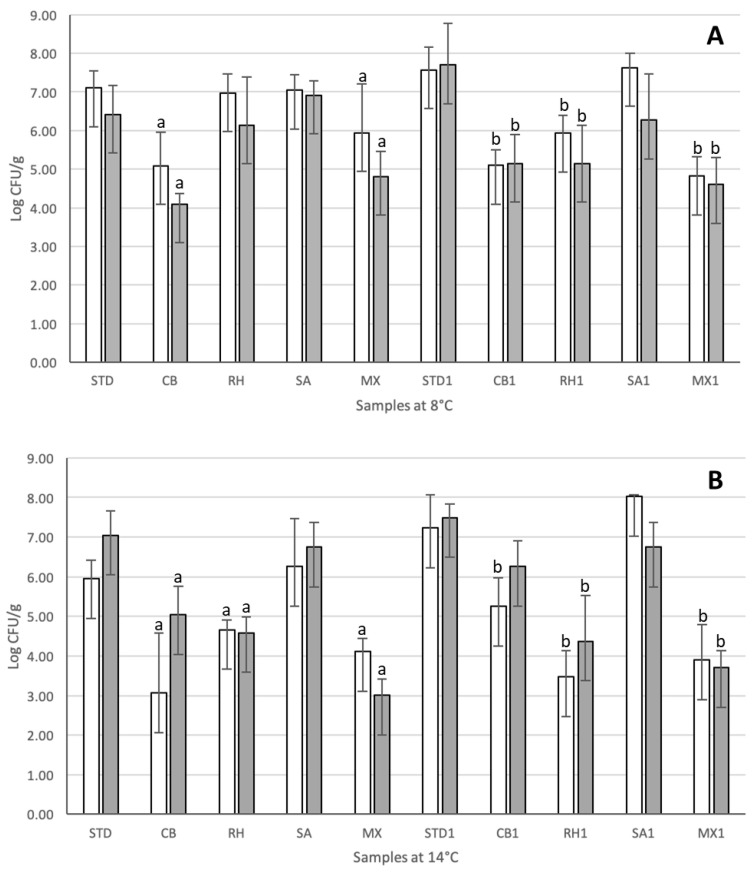
Viable counts on VRBA (white bars) and *Pseudomonas* Agar (grey bars) of cheese stored at 8 °C (**A**) and 14 °C (**B**). STD: control; CB: *C. maltaromaticum* CNB06; RH: *Lcb. rhamnosus* RH05; SA: *Llb. sakei* LSK04, MX: mix of the three adjunct strains. The number 1 after the culture code indicates the strains intentionally spiked with spoilage bacteria. Error bars indicate the standard deviations and letters above the bars indicate the significant difference of treatments (n = 3 and *p* < 0.05) when compared to the relevant controls (a in non-spiked samples and b in artificially contaminated cheeses).

To study the effect of the storage temperature on the spoilage microbiota development during shelf life and to assess the spoilage mitigation in fresh cheese, a trial in an industrial plant was performed, reproducing for the shelf life period at 8 °C, the average temperature of domestic refrigerators [32], and a thermal abuse at 14 °C. The microbiological plate counts on selective media for *Enterobacteriaceae* and *Pseudomonas* from three independent productions for each condition are reported in Figure 2.

For what concerns the STD samples, no evident effect of temperature was observed in the plate counts on the two selective media, despite the lower pH values in control samples at 14 °C than those stored at 8 °C (5.3 and 5.9 respectively). This pH reduction was associated with an increase in lactic acid bacteria counts when samples were stored at 14 °C.

**Figure 3:**
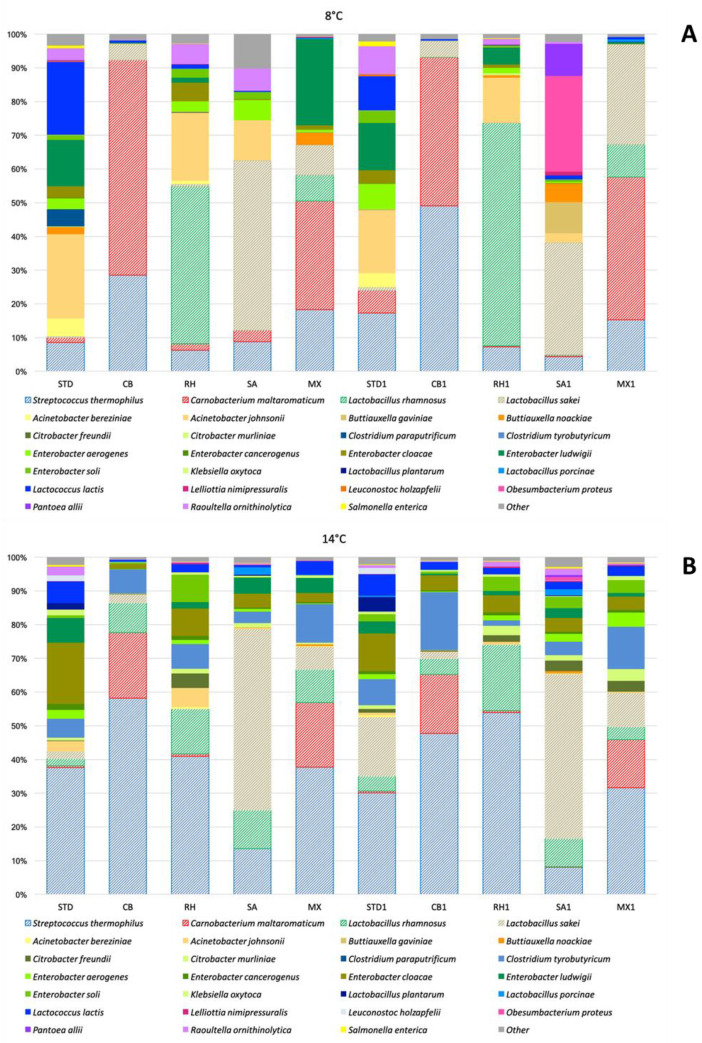
Relative abundance of taxonomic units in cheese samples stored at 8 °C (**A**) and at 14 °C (**B**). STD: control; CB: *C. maltaromaticum* CNB06; RH: *Lcb. rhamnosus* RH05; SA: *Llb. sakei* LSK04, MX: mix of the three adjunct strains. The number 1 after the culture code indicates the strains intentionally spiked with ten strains of spoilage bacteria. Dashed bars indicated the species used as starter or adjunct cultures.

When cheese samples inoculated with the adjunct cultures were analyzed applying the cultivation-dependent approach, the effect on the spoilage microbiota was observed at both temperatures and in presence or absence of spiking (Figure 2). At 8 °C, a significant reduction of spoiling bacteria when compared to STD control samples, made with the starter *S. thermophilus* alone, was observed. Counts on VRBA decreased of 2.0 Log CFU/g and *Pseudomonas* agar of 2.3 Log CFU/g CB cheeses produced with *C. maltaromaticum* CNB06 alone and more than 1 Log CFU/g on both media was obtained in MX cheeses produced with a mixed combination of the three protective strains, (Figure 2A). In cheeses intentionally spiked with the mix of isolated spoilage strains, this effect was higher, being the mitigation effect evident not only in CB1 and MX1 samples but also in RH1 samples inoculated with *Lcb. rhamnosus* RH05 respect to STD1 cheese made without adjunct cultures (Figure 2A). Similarly, in cheeses stored at 14 °C, *Lcb. rhamnosus* RH05, *C. maltaromaticum* CNB06, and the mix of the three adjunct cultures significantly decreased the counts of spoilage bacteria in both natural and spiked treatments (Figure 2B). Samples CB, MX, CB1, RH1, and MX1 at 8 °C and CB, RH, MX, RH1, and MX1 at 14 °C did not present evident sign of spoilage, discoloration, and package blowing. Metagenomic results in terms of relative abundance of taxonomic units at species level for cheese samples with protective cultures stored at 8 and 14 °C is shown in Figure 3.

High-throughput 16S rRNA gene sequencing, applied to assess mainly the changes induced by LAB cultures on the microbial community of cheeses in the two experimental conditions, resulted in 14,756,157 raw reads that were automatically filtered to 13,565,705 by the Miseq software. After the Flash step, the extended frags were 9,346,660 with an average length of 465 bp (± 6.6) bp. Biological replicates were pooled to a total of 7,578,763 reads and 542 OTUs were obtained after OTU picking and filtering steps. The 16S rRNA gene metabarcoding done on one independent production gave a total of 465,040 reads for STD at 8 °C and 413,106 for STD at 14 °C with an average length of 465 bp (± 6.6). The analysis revealed 382 different OTUs in STD at 8 °C and 411 in STD at 14 °C. In terms of phyla, the bacterial community of STD at 8 °C was constituted by approximately 62% of *Proteobacteria* and 35% of *Firmicutes*, while in STD at 14 °C *Proteobacteria* decreased to 40% and *Firmicutes* raised to 59%. The dominant species detected in STD at 8 °C are reported in Figure 3A: Gram-positive species were the starter culture *S. thermophilus* and *Lactococcus lactis*, which accounted respectively to 8% and 22% of detected amplicons. Spoilage bacteria represented the dominant group, being *Acinetobacter johnsonii* the 25% and members of the *Enterobacter* genus the 20% of the total microbial community. At 14 °C (Figure 3B), the proportion of single species changed: *Lactococcus lactis* decreased (7%), *S. thermophilus* increased (38%), and *L. plantarum*, *Llb. Sakei*, and *Lcb. rhamnosus* were detected. The spoilage microbiota did not significantly change its composition. In none of the analyzed samples, members of the genus *Pseudomonas* were detected, demonstrating the effect of MAP in controlling this bacterial group. As a matter of fact, positive counts found on *Pseudomonas* agar base were mostly characterized as members of the *Enterobacteriaceae* family.

**Figure 4:**
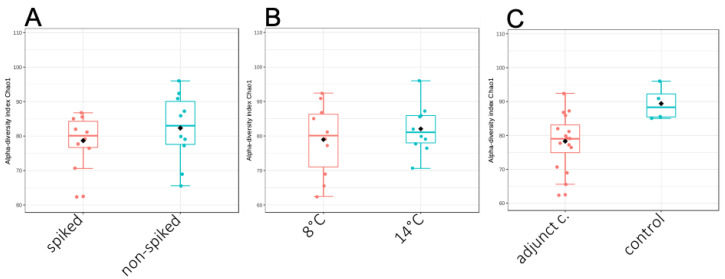
Alpha diversity of cheese samples comparing spike and non-spiked samples (**A**) cheese stored at 8 and 14 °C (**B**) and cheese with and without the adjunct cultures (**C**). Alpha-diversity measured by detected species and Chao1 diversity Index is plotted. The line inside the box represents the median, while the whiskers represent the lowest and highest values within the 1.5 interquartile range. Outliers and individual sample values are shown as dots.

According to the meta-barcoding approach, the spread on the cheese surface of the ten spiking strains did not affect the natural species composition of the analyzed samples; only the spiking species *Obesumbacterium proteus* was detected in low amounts in SA1 samples. Consistently, the alpha-diversity index of spiked and non-spiked samples was not statistically different, as shown in Figure 4A. No difference in the alpha-diversity index of samples incubated at 8 and 14 °C was also observed (Figure 4B), while the use of adjunct cultures had statistically significant effect (*p* < 0.05) on cheese microbiota at both temperatures of storage (Figure 4C).

The three species added as adjunct cultures were able to colonize the cheese, being detected as dominant populations either when used as single strain or mixed culture (Figure 3). At 8 °C, the most effective strain was *C. maltaromaticum* CNB06, which reduced the number of amplicons related to Gram-negative bacteria below 2% as well as the strains mix, particularly in the spiked samples, confirming the plate counts data (Figure 2). At 14 °C, all the tested adjunct cultures reduced the prevalence of Gram-negative species and in treatments with adjunct cultures MX, CB1, and MX1 at 14 °C also an increase of *C. tyrobutyricum* was observed.

### 3.4. Effect of Adjunct Cultures on the Functional Profiling of Cheese Microbiome

**Figure 5:**
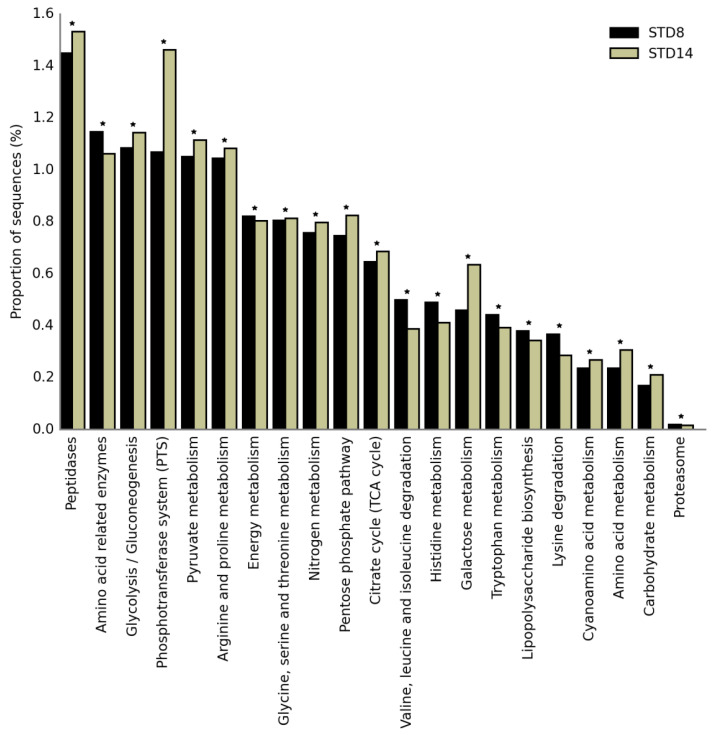
Predicted functional metagenomes of fresh cheeses stored at 8 °C (black) and 14 °C (grey). * statistically significant difference (*p* < 0.05).

The functional profiling of cheese microbiome, based on functionality prediction, revealed significant differences in KEGG orthologues when the control cheese samples, stored at 8 and 14 °C, were compared to samples with adjunct cultures. Among the affiliated KEGG pathways that reached a statistically significance (*p* < 0.01), 21 pathways were selected, with relevance for the bacterial metabolism in cheese as reported in Figure 5.

In cheese stored at 14 °C, genes coding for carbohydrate metabolism, including sugar phosphotransferase system (PTS), galactose metabolism, glycolysis, and pentose phosphate pathway increased if compared to samples at 8 °C. Differently, at the highest temperature, a significant reduction of genes involved in the amino acids metabolism and degradation was observed.

**Figure 6:**
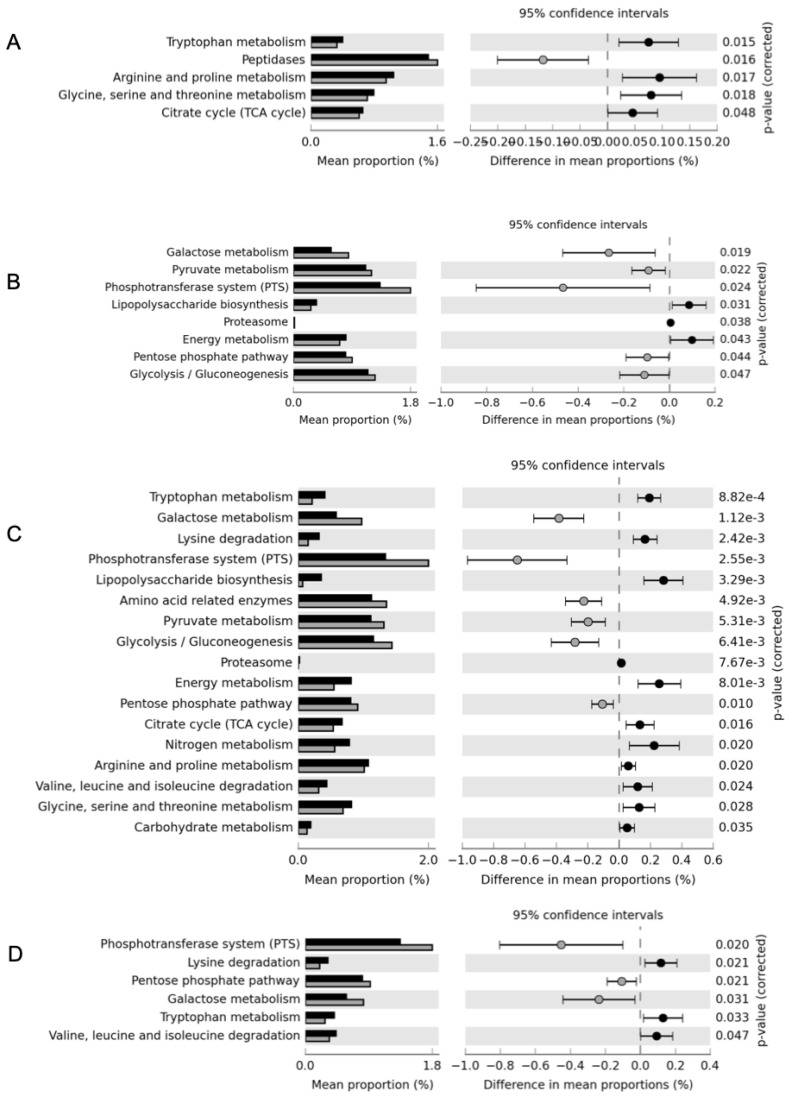
Extended error bar profile in predicted functional metagenomes of fresh cheese in presence of adjunct cultures (grey) *Llb. sakei* LSK04 (**A**), *Lcb. rhamnosus* RH05 (**B**), *C. maltaromaticum* CNB06 (**C**), and mix of adjunct cultures (**D**) in comparison with the standard production (black). Significant *p*-values are reported on the right.

The effect on bacterial metabolism produced by the different protective strains in terms of functional categories is reported in Figure 6. In cheese made with *C. maltaromaticum* (Figure 6C), 17 out of 21 affiliated KEGG pathways were modulated; in particular, sugar metabolism pathways (galactose metabolism, PTS system, glycolysis, pentose phosphate pathway, and pyruvate metabolism) increased, while amino-acids metabolism (tryptophan; lysine; arginine, and proline; valine, leucine, and isoleucine; glycine, serine, and threonine), together with lipopolysaccharides biosynthesis and nitrogen metabolism decreased. Similarly, *Lcb. rhamnosus* RH05 (Figure 6B) had an effect on the cheese microbiome, increasing the energetic metabolism pathways, including galactose metabolism, pyruvate metabolism, PTS system, pentose phosphate pathway, and glycolysis. Although *Llb. sakei* LSK04 (Figure 6A) and the mix (Figure 6D) showed a more limited effect than the other two single protective strains, the trend was similar and a reduction in amino acids metabolism, including tryptophan, lysine, arginine and proline, glycine, serine, threonine, and branched chain amino acids, was observed.

## 4. Discussion

The increasing demand of low-salt and low-fat cheese requires new hurdle technologies approaches to reduce the spoilage microbial communities along the shelf life, particularly by psychotropic Gram-negative bacteria [33,34] that can also trigger antimicrobial resistances [35]. To address this issue, this study focused on the characterization of the spoilage microbiota of these dairy products and the appraisal of lactic acid bacteria adjunct cultures in limiting the outgrowth of these undesirable microorganisms and consequently cheese defects.

The composition of the microbial community of a low salt fresh cheese (0.65% salt, pH 5.8) was examined after an extended shelf life, when defects such as gas production, surface discoloration, and off-flavors are usually present. This product was examined twice: first, samples collected in two different seasons, and then the samples used as control during the industrial trial. In the 30 cheese samples, selected after the end of the shelf life from both summer and autumn periods of production, psychotropic Gram-negative bacteria, *Enterobacteriaceae* and *Pseudomonas*, represented the dominant population of spoiled cheeses, as already observed [33,34].

When bacterial strains were isolated and identified, *Obesumbacterium proteus* was the most frequent, accounting for more than 40% of isolates in both seasons; this microorganism is usually recognized as a brewery contaminant [36] but was also found in cheese [37]. Other bacterial spoilers found at a lower frequency belonged to *Aerococcus*, *Enterococcus*, and *Pseudomonas*. *Hafnia alvei*, a well-known component of smear-ripened cheese [38] that can affect aromatic features by producing sulfur compounds and free amino acids, was also found. When the microbiota of fresh cheese was investigated by DNA metabarcoding approach, the Gram-negative spoilage microbiota represented the 90% of the total microbial community, with the dominance of *Acinetobacter* and *Enterobacter*. Differently from what is observed in other studies [39,40], *Pseudomonas* were not found in the control samples, neither in the other samples using DNA metabarcoding analysis, confirming the effectiveness of the MAP. Although defects were still present, control cheeses stored at 14 °C presented a higher diversity in terms of OTU, with a decreased proportion of Enterobacteriaceae and an increased prevalence of *Firmicutes* (40%), mainly *S. thermophilus* and *Lactobacillus* species. This can be explained by a more permissive storage temperature that can engage competitive interactions between the spoilage microbiota and LAB. The effect of temperature is also highlighted by the predicted functional profiling of cheese microbiome where samples stored at 14 °C presented a significant reduction in the metabolic pathways, such as those involved in amino acid degradation, that can lead to unpleasant organoleptic defects.

With the aim of reducing the identified spoilage microbiota in industrial manufacturing, it was necessary to test the bio-preservative effect of different protective cultures in a fresh cheese through a combination of culture-based and next generation sequencing approaches. Differently from previous studies, where the effect of commercial or selected protective cultures was tested directly during cheese manufacturing [41,42], adjunct LAB cultures were firstly selected, which could specifically inhibit the spoilage microbiota from the cheese under examination and subsequently trialed in industrial cheese manufacturing. The preliminary screening in plate and in cheese models allowed to select three LAB strains, *Llb. sakei* LSK04, *Lcb. rhamnosus* RH05, and *C. maltaromaticum* CNB06 that reduced of almost 3 Log CFU/g *Enterobacteriaceae* and *Pseudomonadaceae* populations at 8 and 14 °C in a cheese model. The scale-up at industrial level was made adding the three selected strains, alone or in pool, directly in milk for cheese production and studying the development of spoilage during the storage at 8 and 14 °C under MAP, a technology widely used to improve the shelf life of fresh dairy products, limiting the growth of pathogenic and alterative microorganisms [34,43].

At 8 °C, the temperature most frequently detected in consumers’ refrigerators (EFSA, 2008), all protective strains were able to grow in the cheese matrix, demonstrating a good adaptation to the substrate. *C. maltaromaticum* CNB06 and, consequently the mixed culture, showed the highest protective action, reducing of more than 2 Log CFU/g the rate of undesired microorganisms respect to the control cheese. In addition, in samples where a mix of *Enterobacteriaceae* and *Pseudomonas* was intentionally spiked by spraying on cheese surfaces, *C. maltaromaticum* CNB06 confirmed its inhibitory effect, lowering the spoiling bacteria between 2–3 Log CFU/g. Food-isolated *C. maltaromaticum* has been extensively studied for its inhibitory capacity due to bacteriocin production, particularly against *Listeria monocytogenes* [44,45]; moreover, this bacterium plays a positive role in soft cheese making thanks to the ability to be acid tolerant without affecting the starter activity during coagulation, and conferring also a pleasant malty aroma to the final product [33,42,46]. Additionally, in spiked samples, *Lcb. rhamnosus* RH05 showed a significant reduction of the spoilage microbiota. In case of thermal abuse (14 °C), the protective effect of selected strains was even higher due to an improved growth of LAB at this temperature with consequent pH lowering to average values below 5.0. In this condition, the efficacy of the protective treatments, with a 2 Log CFU/g reduction, was demonstrated for *C. maltaromaticum* CNB06, *Lcb. rhamnosus* RH05, and the combination of the three strains.

When the DNA metabarcoding data were analyzed for the cheese samples with the addition of protective cultures, the results obtained by the cultivation-dependent approach were confirmed. The inhibitory potential at 8 °C of *C. maltaromaticum* CNB06 was demonstrated; in both CB and CB1 cheese samples, the Gram-negative population was below the 5% of the total OTUs, being the microbiota mainly composed by *C. maltaromaticum*, *S. thermophilus*, *Llb. sakei*, and *L. lactis*. This proved that *C. maltaromaticum* CNB06 was well adapted to fresh cheese and was able to colonize the dairy environment at refrigeration temperature even when directly added to milk in the vat for cheese making. Spanu et al. [42] previously confirmed the protective potential of this psychrophilic microorganism to control the growth of spiked *Pseudomonas* spp. when inoculated on the surface of ricotta fresca cheese and supported previous studies conducted on naturally contaminated sheep’s milk ricotta fresca [33]. Moreover, *C. maltaromaticum* demonstrated to increase the sugar metabolism in cheese at the end of shelf life despite the decrease of amino acids and lipopolysaccharides metabolisms; this is in line with the high numbers of carbohydrate transport and metabolism genes found on the chromosome of *Carnobacterium* spp. previously sequenced [47]. In addition, the inhibitory capacity of *C. maltaromaticum* seems to be correlated to bacteriocins synthesis but also to the production of formate and acetate organic acids [48]. Therefore, its growth is not associated to high pH variations at the end of the shelf life respect to the standard sample (5.56 versus 5.76).

When *Lcb. rhamnosus* RH05 was added as protective culture, more than 50% of the taxonomic units were assigned to this LAB species at 8 °C and 30% at 14 °C. In spiked RH1 samples, the spoilage microbiota accounted for less than 30%, being represented by *Acinetobacter* and *Enterobacter*. The protective effect of this strain, although less effective than *C. maltaromaticum* CNB06, allowed to reduce unwanted microorganisms and delay the product spoilage. *Lcb. rhamnosus* RH05 in a blend with *L. plantarum* and *E. faecium* strains had a low mitigation activity against *Pseudomonas* in ricotta fresca cheese [33]. Differently, when the same strain was tested with the solely *L. plantarum* in burrata cheese [49], it slowed the growth of staphylococci, coliforms, and *Pseudomonas* spp., especially in early storage, increased the product shelf life of three days, and improved the flavor. Furthermore, the role of *Lcb. rhamnosus* as a promising protective culture was widely demonstrated in dairy products [50,51] and also in food coating applications where cell-free supernatant of *Lcb. rhamnosus* were effective in inhibiting *E. coli*, *L. monocytogenes*, *S. aureus*, or *Salmonella* Typhimurium when used with whey protein films in packaging [52].

Although the cultivation-based data did not show a significant reduction of the spoilage counts, metabarcoding outcomes in samples stored at both temperatures, when *Llb. sakei* LSK04 was used as the only adjunct culture, indicate that this bacterium colonized the cheese during the shelf life. According to our experiments in fresh cheese, *Llb. sakei* LSK04 was not a good candidate for its inhibition capacity among the tested microorganisms, since, also in condition of thermal abuse, the strain was not able to provide a good level of protection, even if prevailed in SA and SA1 samples as taxonomic unit on the total OTU abundance.

*Latilactobacillus sakei* is more frequently used as a protective agent in meat and meat products to control *Listeria monocytogenes* and other pathogenic bacteria [53,54] thanks to its ability to grow in cold storage conditions and driving, at the same time, the fermentation process. When this species is forced to grow in a different food environment, such as cheese, it can sometimes vary its own efficacy [55].

When the three adjunct cultures were evaluated as a pool, positive results were obtained at 8 and 14 °C mostly due to the presence of *Carnobacterium*, but also to the synergic effect generated by bacteriocins production by the three bacterial strains. LAB mixed cultures often demonstrated to exert a highest protective action without affecting the cheese making and the final organoleptic properties [14,15,56]. As a general observation, the use of adjunct cultures significantly shapes the cheese microbiota, by reducing the alpha-diversity index, as shown in Figure 4. This is the consequence of the inhibitory activity played by the studied LAB against Gram-negative bacteria.

In this study, we have analyzed the same samples using different approaches, plate counts, and OTU detection by metabarcoding. Although these two techniques are known to provide a different estimation of the bacterial community composition [57], the results obtained in this study are consistent, showing that the decrease of 2-log CFU on selective media corresponds to approximately a 90% reduction of the corresponding spoiling population in OTU analysis.

The use of bacterial cultures as protection against spoiling and pathogenic microorganisms represents a natural and affordable intervention in the food industry. Our work provides additional data supporting the efficacy of selected adjunct cultures in limiting the Gram-negative spoilage populations in fresh cheese by reshaping the cheese microbiome.

## Figures and Tables

**Table 1:** List of bacterial strains used in this study.

Strain	Use
*Streptococcus thermophilus* ST022	Starter culture
*Lacticaseibacillus rhamnosus* UC8490	Adjunct culture
*Lactiplantibacillus plantarum* UC8491	Adjunct culture
*Latilactobacillus curvatus* UC8266	Adjunct culture
*Latilactobacillus curvatus* UC8265	Adjunct culture
*Lacticaseibacillus casei* UC8561	Adjunct culture
*Ligilactobacillus acidipiscis* UC8115	Adjunct culture
*Lactiplantibacillus plantarum* LP48	Adjunct culture
*Lactiplantibacillus plantarum* LP52	Adjunct culture
*Latilactobacillus sakei* LSK04	Adjunct culture
*Lacticaseibacillus casei* LC10	Adjunct culture
*Lacticaseibacillus rhamnosus* LRH05	Adjunct culture
*Carnobacterium maltaromaticum* CNB04	Adjunct culture
*Carnobacterium divergens* CNB05	Adjunct culture
*Carnobacterium maltaromaticum* CNB06	Adjunct culture
*Obesumbacterium proteus* UC7452,	Indicator/Spiking
*Obesumbacterium proteus* UC7453	Indicator/Spiking
*Obesumbacterium proteus* UC7454	Indicator/Spiking
*Obesumbacterium proteus* UC7456	Indicator/Spiking
*Obesumbacterium proteus* UC7457	Indicator/Spiking
*Obesumbacterium proteus* UC7459	Indicator/Spiking
*Pseudomonas libanensis* UC7310	Indicator/Spiking
*Pseudomonas fragi* UC7455	Indicator/Spiking
*Pseudomonas gessardii* UC7458	Indicator/Spiking
*Aerococcus urinaequi* UC7460	Indicator/Spiking

**Table 2:** Prevalence of bacterial species isolated from spoiled cheeses and time of production.

Species	Frequency of Isolation (%)	Production Period
*Obesumbacterium proteus*	42.6	summer and autumn
*Aerococcus urinaeequi*	8.8	summer
*Enterococcus faecalis*	6.4	summer
*Latilactobacillus graminis*	5.8	summer
*Pseudomonas brennerii*	5.3	autumn
*Carnobacterium gallinarum*	4.7	summer
*Pseudomonas libanensis*	4.7	summer
*Leuconostoc pseudomesenteroides*	4.0	summer
*Hafnia alvei*	2.9	autumn
*Pseudomonas fragi*	2.3	summer
*Pseudomonas psychrophila*	1.8	summer
*Pseudomonas gessardii*	1.7	autumn
*Exiguobacterium acetylicum*	1.2	summer
*Macrococcus caseoliticus*	1.2	summer
*Staphylococcus vitulinus*	1.2	summer
*Lacticaseibacillus paracasei*	1.2	summer
*Pseudomonas proteolytica*	1.2	autumn
*Serratia liquefaciens*	1.2	summer
*E. pseudoavium/devriesei*	0.6	summer
*Lactiplantibacillus paraplantarum*	0.6	summer
*Leuconostoc mesenteroides*	0.6	summer
